# Satisfaction with pandemic management and compliance with public health measures: Evidence from a German household survey on the COVID-19 crisis

**DOI:** 10.1371/journal.pone.0281893

**Published:** 2023-02-21

**Authors:** Philipp Jaschke, Sekou Keita, Ehsan Vallizadeh, Simon Kühne

**Affiliations:** 1 Institute for Employment Research (IAB), Nuremberg, Germany; 2 University of Bamberg, Bamberg, Germany; 3 Bielefeld University, Bielefeld, Germany; Universitatsklinikum Schleswig Holstein Campus Lubeck, GERMANY

## Abstract

We study how satisfaction with government efforts to respond to the COVID-19 crisis affects compliance with pandemic mitigation measures. Using a novel longitudinal household survey for Germany, we overcome the identification and endogeneity challenges involved in estimating individual compliance by using an instrumental variable approach that exploits exogenous variation in two indicators measured before the crisis: political party preferences and the mode of information measured by the frequency of using social media and reading newspapers. We find that a one unit increase in subjective satisfaction (on the 0-10 scale) improves protective behavior by 2-4 percentage points. Satisfaction with the government’s COVID-19 management is lower among individuals with right-wing partisan preferences and among individuals who use only social media as an information source. Overall, our results indicate that the effectiveness of uniform policy measures in various domains, such as the health system, social security or taxation, especially during pandemic crises, cannot be fully evaluated without taking individual preferences for collective action into account.

## 1 Introduction

The COVID-19 pandemic has claimed the lives of more than 6 million people (as of May, 2022). Governments around the world have implemented a vast number of management strategies and strict public health measures, such as lockdown and social distancing. However, in democratic societies, governments cannot enforce them by means of coercion or by monitoring and controlling the whole society is neither feasible nor desirable [1]. Moreover, the effectiveness, e.g., lower transmission rate of infection, and the efficiency, e.g., lower monitoring and enforcement costs, of these policies depend largely on behavior adaption and on individual commitment and careful preparedness [2–4]. From an economic point of view, a key component of strategic behavior is that individual performance and motivation depend strongly on personal attitudes and subjective well-being preferences and the expected value of a specific action. Such attitudes and subjective perceptions are considered to be important in various domains such as in the context of job-satisfaction and occupational commitment [5] and of happiness and tax compliance [6].

From a health policy perspective, uniform health measures that treat all people symmetrically can bear larger social and economic costs compared to targeted measures that differentiate between risk and non-risk groups [4, 7, 8]. This is particularly the case whenever the short-term costs, e.g., imposing strict lockdowns and social distancing, are perceived more directly than medium- and long-run benefits, e.g., well-functioning healthcare systems. As a consequence, a gap may arise between the individual incentives to comply with public health measures and the socially desired level of compliance. This study addresses the question of whether increased satisfaction with government pandemic management induces more compliance with public health measures, thereby reducing the gap between individual’s perceived costs of compliance and their perceived advantages of compliance for society.

Specifically, the cost of public health measures in the wake of the COVID-19 pandemic was not equally distributed [9]. Therefore, clear communication and consultation of policy measures would enhance the understanding of policy goals. This could raise satisfaction with government pandemic management and, in turn, increase both individual protective behavior (e.g., wearing face masks or social distancing) and improve health outcomes. Furthermore, understanding the effect of satisfaction with government pandemic management on compliance with health policy measures is highly relevant for a number of reasons. In the context of medical treatment, patients’ degree of satisfaction with healthcare services contributes to their commitment to and compliance with recommended treatments [10, 11]. Moreover, policies induce social action, which entails costs for some individuals to achieve positive outcomes for society. To assess the net effects of policies for society, policymakers need to take into account individual’s preferences, i.e. their perceptions regarding advantages or values associated with the success of the policy [12].

Compelling evidence on the relationship between individual satisfaction with government management and compliance with public health measures in the context of pandemics and health crises is lacking. The aim of this study is therefore to investigate whether and how subjective satisfaction with the government’s pandemic management explains individual compliance with protection and health measures. To this end, we conduct a multivariate regression analysis combining original data collected both before and during the pandemic.

## 2 Materials and methods

The survey used in our study was approved by the ethics committee of Bielefeld University (application number: 2022–040). Statistical analyses were performed with STATA 17.0 (StataCorp LLC, College Station, TX, USA). Syntax files for replication of results are provided in Stata Do-File format at https://doi.org/10.17605/OSF.IO/HC2MD.

### 2.1 Data sources

Our analysis builds on data derived from the SOEP-CoV project [13]—a collaboration of Bielefeld University and the SOEP research infrastructure unit at the German Institute for Economic Research (DIW Berlin). SOEP-CoV is a COVID-19-related, probability-sample survey extension of the SOEP, one of the largest and longest-running panel surveys worldwide [14]. SOEP conducts annual interviews with individuals in private households in Germany. It consists of multiple random (enlargement/refreshment) sub-samples, that have been drawn throughout the years [15]. Applying survey weights, SOEP data is representative for the German private household population both in cross-sectional as well as in longitudinal analysis settings.

SOEP-CoV is funded by the German Federal Ministry of Education and Research (BMBF) as part of its “call for proposals for research on COVID-19 in the wake of the Sars-CoV-2 outbreak.” The Institute for Employment Research (IAB) participated in the project as a cooperation partner for the IAB-SOEP Migration Sample (subsamples M1, M2; see [16]) and the IAB-BAMF-SOEP Survey of Refugees (subsamples M3, M4, M5; see [17, 18]). The SOEP-CoV data is officially available to the scientific community with the regular rollout of the SOEP-Core data v37 (DOI: 10.5684/soep.core.v37eu) by the SOEP Research Data Center. Information on data access can be found at [19]. Note that for access to regional data (residence places of survey respondents), an expanded data distribution contract is required (for more information refer to [20]).

The SOEP-CoV survey covers two waves and was administered in the computer-assisted telephone interview (CATI) format between April and June 2020 (wave 1) and January and February 2021 (wave 2). It is based upon a random sample of private households that previously participated in the 2018 and 2019 wave of the SOEP. In each household, one individual (18+) was interviewed. The data were collected from 6,700 respondents interviewed during the COVID-19 pandemic in 2020 (wave 1), 6,000 of whom could be reinterviewed a second time in early 2021. In wave 1, households were divided into 9 tranches, each representing a random subsample and interviewed consecutively throughout the survey phase. For an overview of the topics and a detailed explanation of the methodology, see [13].

In this paper, we use data from tranches 2–4 of SOEP-CoV wave 1 and the entire data of wave 2. The reason for dropping the first tranche of about 1,700 individuals is that selected components of compliance measures that we employ for our dependent variable were not elicited. Additionally, we lose about 1,500 observations from tranches 5–9 since our main explanatory variable “satisfaction with the German Federal government’s crisis management” was not part of the questionnaire. The results are robust due to the random design of each tranche. Taken together, our estimation sample covers 6,149 person observations surveyed in wave 1 between April and May 2020 (3,390 person observations) and in wave 2 between January and February 2021 (5,820 person observations). This gives us both variation in the prevailing regional infection situation and in the infection prevention measures either recommended or even mandated by the government at the time of interviews; 75 percent of the interviews in wave 1 of our working sample were conducted in April, during the period in which far-reaching health-protective measures have just been implemented in Germany. Most of these measures were still in place during the field phase of the second wave or were reintroduced due to renewed increases in infection rates after the temporary easing in the summer 2020.

The survey data cover detailed information on respondents’ current health, social and employment situation, economic risks, values and preferences, and much more. We drop 19 respondents from wave 1 and 36 respondents from wave 2 who did not answer the question regarding satisfaction with the government’s crisis management. In case of missing values in the remaining control variables, we control for a missing category using a dummy variable. The proportion of missing values can be inferred by comparing the values in columns titled N in Table 2, and is not more than 4% for any control variable. Given the panel structure of the data, we combine information from the COVID-19 survey with that from the regular SOEP study before COVID for each participant. Specifically, we use the SOEP-Core (v36) data collected between August and December 2019, i.e., closely before the COVID-19 crisis started. All individuals in our sample as described above also participated in the 2019 panel survey. Taken together, this provides a rich data set that includes both pre- and post-crisis information for each individual.

We complement the SOEP survey data by including aggregate data on the unemployment rate and log population density per square kilometer (both measured at March-31–2020) in the municipality of residence [21, 22] and the COVID-19 incidence rate (measured per 100,000 residents in the last 7 days before the interview, provided by the Robert Koch Institute and downloaded from [23]) at the district level. In addition, we use an indicator of available inpatient medical care capacity (measured by the number of hospital beds available for acute care patients in 2016 per 1,000 residents [24]) at the district level. This proxy for regional health system capacity is relevant to avoid omitted variable bias. For example, individuals in regions with high hospital capacity might feel safer from the virus and therefore be less compliant while being more satisfied with the government.

### 2.2 Variables

To investigate our main research question, we use the answers to several survey items. First, we use the answer to the question *“How satisfied are you with the coronavirus crisis management by [authority X]?”* as our main explanatory variable. The answer categories included the main authorities at the federal and local levels, such as *“the federal government”* and *“the government of the federal state (Bundesland) where you live”* on a scale of 0 (not satisfied at all) to 10 (very satisfied).

Second, for our outcome variable, we exploit different items measuring an individual’s protective behavior. Particularly, we use answers to the following question: *“There are various recommendations for how to behave in everyday life and in public to prevent the spread of the novel coronavirus. If you think back over the last seven days, have you …”*. This question included up to ten answer items. Two of these answer items were available in a few tranches only: the item *“regularly aired out the living areas of your home?”* was asked in tranche 1 of wave 1 only and the item *“worn a protective mask when shopping or using public transport?”* in tranches 2–4 of wave 1 only and wave 2. Therefore, in order to have a consistent set of individual compliance measures over the survey period, we exclude the item *“regularly aired out the living areas of your home?”* by restricting our final working sample to tranches 2–4 of wave 1 and wave 2 (see section 2.1 above). We calculate our main compliance index as the mean over the 9 items as depicted in Table 1.

**Table 1:** Compliance components behind the main outcome index.

There are various recommendations for how to behave in everyday life and in public to prevent the spread of the novel coronavirus. If you think back over the last seven days, have you …
• avoided contact with elderly, very old or chronically ill people?
• refrained from using public transport?
• refrained from traveling, even within Germany?
• worn a protective mask when running errands or on public transport?
• avoided shopping at peak times?
• stayed away from crowds?
• kept distance from people who have a cough, cold or fever?
• avoided touching such as shaking hands or hugging?
• washed your hands regularly (at least 20 seconds with soap and water)?

Source: Own translations from German SOEP-CoV questionnaire to English [25]. Multiple answers possible.

Finally, to assess the causal effect of satisfaction on compliance, we utilize two measures from the pre-COVID longitudinal SOEP wave 2019 to implement an instrumental variable estimation strategy. First, we use the question on whether participants lean toward any political party. The response items for this question included the leading political parties across the political spectrum in Germany, such as the Social Democratic Party (SPD), the conservative Christian Democratic Party (CDU/CSU), the left-wing party (Die Linke) and the right-wing party (Alternative for Germany [AfD]). Second, we use the question regarding what people do in their leisure time. There were 11 response items, such as *“contacting friends or relatives”* or *“watching TV or movies”*, that respondents could report engaging in at the following frequencies: “daily”, “at least once per week”, “at least once per month”, “seldom”, and “never”. We use the two items *“reading (daily) newspapers (including digital newspapers)”* and *“using online social networks/chat services (e.g., Facebook/Instagram/Twitter/WhatsApp)”* and respectively code the answers “daily” and “at least once per week” as 1 and the remaining answers as 0. Then, we generate a factor variable with four values according to the possible combinations of newspaper reading and social network usage.

Given that both blocks (political party preferences and media usage) were asked in 2019, we use them as separate instrumental variables for our main independent variable “satisfaction with the government’s crisis management”.

### 2.3 Analytical approach

We conduct our empirical analysis using multivariate regressions at the individual level. Specifically, the estimation equation relates the compliance *C* of individual *i* living in region *r* and interviewed in calendar week *t* with the degree of satisfaction *S* of individual *i* with the crisis management of the federal government regarding the COVID-19 mitigation measures:

$$\begin{array}{c} C_{i,r,t}=\alpha_{1}+\beta_{1}S_{i}+\beta_{2}X_{i}+\beta_{3}R_{d,m}+\delta_{r}+\delta_{t}+\varepsilon_{irt}\;, \end{array}$$ (1)

where **X**_**i**_ is a vector of individual controls and *ε*_*iot*_ is an idiosyncratic error term. Due to data availability, we construct two measures of the regional control variable: **R**_**d,m**_ denotes regional controls at either the district *d* or municipality *m* level. In addition, we add regional government (defined by the Nomenclature of Territorial Units for Statistics 2 [NUTS2]) fixed effects *δ*_*r*_ to control for unobserved time-invariant factors at the aggregated regional level that may affect districts or municipalities (there are 38 NUTS2 regions in Germany). We also include calendar-week fixed effects for the time of the interview **δ**_**t**_ to account for all time-specific, region-invariant variation due to the dynamic nature of the pandemic.

We first use linear regression techniques, both applying the standard OLS estimator and the 2-stage-least-squares (2SLS) instrumental variable (IV) estimator. In section 4.3, we relax the linearity assumption, applying the non-linear Probit-IV estimator.

### 2.4 Endogeneity

A strong correlation between two variables does not necessarily reflect a causal relationship. The correlation could arise if the explanatory variable is correlated with the error term, i.e, the unexplained part of the dependent variable in the multivariate regression model. This situation is broadly referred to as endogeneity between the regressor and the outcome variable. It is a common issue in observational studies and it can be the result of several causes. For example, if attentive and careful individuals would have complied even without the government’s protective measures, reverse causality would lead to a downward bias in the estimation. Moreover, there might also be omitted variable bias: Individuals who perceive themselves to be at high risk, e.g., due to chronic diseases, might be more satisfied with the government interventions and comply more with health protective measures. In presence of such endogeneity issues, standard OLS estimators are biased and estimated coefficients do not reflect causal relationships.

To assess the causal effects of subjective satisfaction on protective behavior, we instrument our main explanatory variable with information elicited before the COVID-19 crisis. With our unique panel data, we are able to use information collected in 2019 about the political preferences of participants in the SOEP-CoV survey. This approach contrasts with that in existing studies, which usually use survey information collected only after the outbreak of the pandemic. Specifically, we include seven dummy variables (with *no party preference* as the reference category) corresponding to the six parties currently represented in the German Bundestag and one collective category, (*others*), for all other parties.

Instrumental variable (IV) estimates are valid under two conditions ([26–28]). The first condition is that the instruments are *relevant* in explaining individuals’ satisfaction with the government’s COVID-19 crisis management. The second condition is that the instruments are not part of structural Eq (1), i.e., they satisfy the so-called *exclusion restriction*. Two assumptions underlie this condition: (i) the instruments (political preferences) should not directly affect compliance and (ii) the instrument should be independent of potential outcomes, conditional on covariates. In the next sub-sections, we discuss the plausibility of these two identifying assumptions.

#### 2.4.1 First condition: Relevance

To evaluate the relevance of our IV, in the online Table A.1 in S1 Appendix, we show the first-stage OLS results. We regress *satisfaction with the government’s crisis management* (on the 0–10 scale) on our seven instruments (with *no party preference* as reference category). This approach is similar to that of [29, Table 2], who test trust in state government with individual dummies for whether the government is represented by the respondent’s own preferred party. Supporters of the ruling grand coalition at that time (SPD and CDU/CSU) are the most satisfied, followed by supporters of the Greens. In contrast, AfD supporters have satisfaction scores that are on average more than 2 points lower than those of the reference group (no party preference). All effects remain highly statistically significant when we include additional individual and regional control variables as well as regional and time fixed effects.

**Table 2:** Descriptive statistics of all variables.

	Sample: SOEP-CoV, wave 1 participants (2020)						Sample: SOEP-CoV, wave 2 participants (2021)					
	Mean	Median	SD	Min	Max	N	Mean	Median	SD	Min	Max	N
**A) Compliance measures**:												
Compliance index, share over 9 measures (0—1)	0.873	0.889	0.144	0.000	1.000	3,390	0.879	0.889	0.161	0.000	1.000	5,820
Avoid contact with high-risk patients (0 no, 1 yes)	0.810	1.000	0.392	0.000	1.000	3,390	0.744	1.000	0.437	0.000	1.000	5,820
Stay away from crowds	0.956	1.000	0.205	0.000	1.000	3,390	0.931	1.000	0.253	0.000	1.000	5,820
Wear masks in shops and public transport	0.632	1.000	0.482	0.000	1.000	3,390	0.968	1.000	0.177	0.000	1.000	5,820
Avoid public transport	0.847	1.000	0.360	0.000	1.000	3,390	0.789	1.000	0.408	0.000	1.000	5,820
Avoid shopping at rush hours	0.837	1.000	0.369	0.000	1.000	3,390	0.797	1.000	0.402	0.000	1.000	5,820
Keep distance from people showing symptoms	0.919	1.000	0.274	0.000	1.000	3,390	0.911	1.000	0.285	0.000	1.000	5,820
Avoid touching, such as handshaking, hugging	0.957	1.000	0.204	0.000	1.000	3,390	0.941	1.000	0.236	0.000	1.000	5,820
Refrain from trips	0.925	1.000	0.263	0.000	1.000	3,390	0.878	1.000	0.328	0.000	1.000	5,820
Wash hands regularly	0.976	1.000	0.155	0.000	1.000	3,388	0.952	1.000	0.213	0.000	1.000	5,820
**B) Satisfaction with COVID-19 crisis management of German Federal Government (0 low—10 high)**	6.815	7.000	2.221	0.000	10.000	3,390	5.797	6.000	2.334	0.000	10.000	5,820
**C) Political party preferences before COVID-19 pandemic**:												
None (0 no, 1 yes)	0.522	1.000	0.500	0.000	1.000	3,390	0.506	1.000	0.500	0.000	1.000	5,820
Social-Democrats (SPD)	0.099	0.000	0.299	0.000	1.000	3,390	0.104	0.000	0.305	0.000	1.000	5,820
Convervatives (CDU, CSU)	0.151	0.000	0.358	0.000	1.000	3,390	0.156	0.000	0.363	0.000	1.000	5,820
Liberals (FDP)	0.023	0.000	0.151	0.000	1.000	3,390	0.023	0.000	0.151	0.000	1.000	5,820
Greens	0.121	0.000	0.326	0.000	1.000	3,390	0.128	0.000	0.334	0.000	1.000	5,820
Far-left (Die Linke)	0.040	0.000	0.195	0.000	1.000	3,390	0.043	0.000	0.203	0.000	1.000	5,820
Far-right (AFD)	0.028	0.000	0.166	0.000	1.000	3,390	0.026	0.000	0.158	0.000	1.000	5,820
Other	0.016	0.000	0.125	0.000	1.000	3,390	0.015	0.000	0.121	0.000	1.000	5,820
**D) Media consumption before COVID-19 pandemic**												
Neither used social media, nor read newspapers	0.055	0.000	0.229	0.000	1.000	3,390	0.051	0.000	0.220	0.000	1.000	5,820
Only used social media	0.229	0.000	0.421	0.000	1.000	3,390	0.224	0.000	0.417	0.000	1.000	5,820
Only read newspapers	0.190	0.000	0.392	0.000	1.000	3,390	0.191	0.000	0.393	0.000	1.000	5,820
Both	0.525	1.000	0.499	0.000	1.000	3,390	0.534	1.000	0.499	0.000	1.000	5,820
**E) Individual controls**:												
Age	53.694	54.000	15.886	18.000	99.000	3,390	54.931	55.000	15.657	19.000	100.000	5,818
Female (0 no, 1 yes)	0.616	1.000	0.487	0.000	1.000	3,390	0.606	1.000	0.489	0.000	1.000	5,820
Child in household (0 no, 1 yes)	0.350	0.000	0.477	0.000	1.000	3,389	0.316	0.000	0.465	0.000	1.000	5,820
Highest education: Lower secondary (0 no, 1 yes)	0.082	0.000	0.275	0.000	1.000	3,390	0.080	0.000	0.272	0.000	1.000	5,820
Post-secondaty, non-tertiary	0.534	1.000	0.499	0.000	1.000	3,390	0.541	1.000	0.498	0.000	1.000	5,820
Bachelor	0.224	0.000	0.417	0.000	1.000	3,390	0.231	0.000	0.421	0.000	1.000	5,820
Master or Doctoral	0.153	0.000	0.360	0.000	1.000	3,390	0.143	0.000	0.350	0.000	1.000	5,820
No migration background (0 no, 1 yes)	0.802	1.000	0.398	0.000	1.000	3,390	0.837	1.000	0.369	0.000	1.000	5,820
Direct	0.143	0.000	0.350	0.000	1.000	3,390	0.115	0.000	0.319	0.000	1.000	5,820
Indirect	0.055	0.000	0.228	0.000	1.000	3,390	0.048	0.000	0.214	0.000	1.000	5,820
Living space per person in HH, sqm	51.152	43.500	29.653	9.500	300.000	3,389	51.928	45.000	29.552	9.500	330.000	5,819
Health satisfaction measured before pandemic (0 low—10 high)	6.709	7.000	2.152	0.000	10.000	3,390	6.702	7.000	2.106	0.000	10.000	5,820
Has been at least partly in home-office during pandemic												
(0 no, 1 yes)	0.243	0.000	0.429	0.000	1.000	3,390	0.228	0.000	0.420	0.000	1.000	5,820
Has been in short-time work during pandemic (0 no, 1 yes)	0.090	0.000	0.286	0.000	1.000	3,390	0.046	0.000	0.210	0.000	1.000	5,820
Occupation before COVID-19 pandemic:												
Analytical non-routine tasks (0 no, 1 yes)	0.222	0.000	0.416	0.000	1.000	3,246	0.215	0.000	0.411	0.000	1.000	5,589
Interactive non-routine	0.088	0.000	0.284	0.000	1.000	3,246	0.088	0.000	0.284	0.000	1.000	5,589
Cognitive routine	0.129	0.000	0.335	0.000	1.000	3,246	0.138	0.000	0.345	0.000	1.000	5,589
Manual routine	0.036	0.000	0.186	0.000	1.000	3,246	0.037	0.000	0.188	0.000	1.000	5,589
Manual non-routine tasks	0.095	0.000	0.294	0.000	1.000	3,246	0.098	0.000	0.297	0.000	1.000	5,589
Not employed	0.429	0.000	0.495	0.000	1.000	3,246	0.425	0.000	0.494	0.000	1.000	5,589
Strength of political party preference												
(1 No or very weak—5 very strong)	2.217	1.000	1.375	1.000	5.000	3,377	2.263	1.000	1.379	1.000	5.000	5,799
Calender weak of interview (1—53)	17.286	17.000	1.513	15.000	22.000	3,390	4.295	4.000	1.106	3.000	7.000	5,820
**F) Regional controls**:												
Unemployment rate in municipality, March-31–2020	5.354	4.959	2.410	0.448	17.469	3,390	5.340	4.942	2.454	0.448	17.469	5,820
Population density of municipality at March-31–2020 (per sqkm)	1,119	644	1,194	12	4,736	3,390	1,102	608	1,192	12	4,736	5,820
Covid-19 incidence rate in district												
(last 7 days before interview, per 100k)	15.833	11.400	16.372	0.000	150.700	3,390	106.606	93.100	56.220	17.300	448.200	5,820
Hospital beds in district per 1,000 inhabitants, 2016	6.182	5.790	3.110	0.000	19.810	3,390	6.149	5.760	3.128	0.000	19.810	5,820

Source: SOEP-CoV waves 1, 2 and SOEP-Core of Socio-Economic Panel (SOEP) (v37). Differences in observations are due to missings for which we control in our main analyses.

#### 2.4.2 Second condition: Exclusion restriction

With regard to the *exclusion* criteria, the panel structure rules out the possibility of simultaneity because the instruments are measured in 2019, i.e, before the COVID-19 pandemic. In other words, knowing that political preferences have been measured in a different survey wave one year before the compliance variables greatly mitigates concerns that the instruments share any causes with the outcome. Furthermore, no obvious reason indicates that party affiliation should systematically and directly impact individual compliance with COVID-19 containment measures through another channel than satisfaction with the government’s COVID-19 management.

Research from the US and Brazil does find a high explanatory power of political party affiliation for compliance with social distancing and individual containment measures; i.e., there is an enormous divergence in such compliance between Republicans and Democrats in the US ([29–32]) as well as supporters and opponents of President Bolsonaro in Brazil [33]. However, while media usage and risk assessment seem to play a role, little is known about the actual mechanisms [30].

The political situation in Germany is different. There is a cross-party consensus on the necessity and usefulness of protection and hygiene measures. Until recently, even the far-right AfD, which is closest on the German political spectrum to the populist Trump and Bolsonaro administrations, supported the extensive hygiene measures recommended by the German government. In an official position paper on the COVID-19 crisis released on April 8 2020, the AfD parliamentary group in the German Bundestag criticized the federal government’s crisis management but only in that the government had allegedly acted too late and instead should have implemented extensive hygiene measures earlier [34]. Point 3 of the paper explicitly calls for special protection of at-risk groups, compliance with hygiene and social distancing rules and consistent wearing of mouth and nose masks to protect others.

However, approximately a week later, after the first phase of relaxation of the crisis measures in Germany, the health policy spokesperson of the AfD parliamentary group stated that a further-reaching relaxation would have been possible with the “instruction to the population to continue to deal with this problem in a disciplined and hygienic manner” [35]. It was only toward the end of May 2020 that the political agenda of the party changed and the AfD parliamentary group filed a lawsuit before the Brandenburg Constitutional Court complaining about the disproportionate impact of the obligation to wear masks on the retail trade [36]. In our data, 75% of respondents in the first wave of SOEP-CoV were interviewed in April and 90% by mid-May, i.e., before the party changed course in its COVID-19 politics. Therefore, we doubt the possibility of a direct effect of political party affiliation on compliance.

## 3 Results

### 3.1 Descriptive analysis

**Fig 1:**
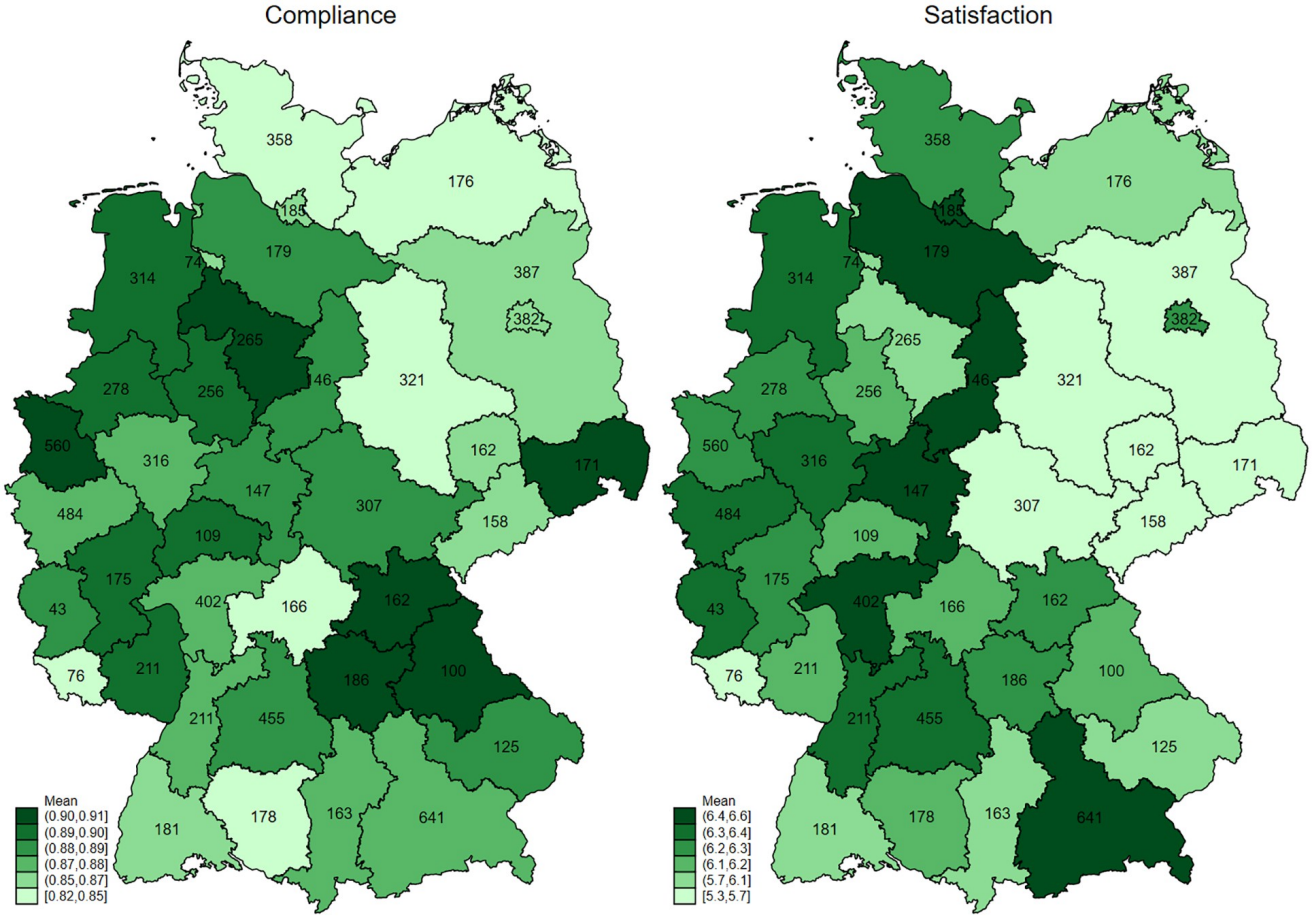
Distribution of compliance and satisfaction over NUTS-2 regions. Source: SOEP-CoV waves 1, 2 and SOEP-Core of Socio-Economic Panel (SOEP) (v37). The figure shows respondents’ mean values of compliance and satisfaction at the NUTS-2 region level. Compliance is calculated as the mean over 9 compliance components (dummy variables) as depicted in Fig 1 and ranges from 0 (does not comply with any measure) to 1 (complies with all measures). Satisfaction with the government’s COVID-19 crisis management ranges from 0 (low)—10 (high). The numbers on the map indicate the observation numbers, aggregated over wave 1 (2020) and wave 2 (2021). Over all NUTS-2 regions, observations add up to 9,210 (3,390 in wave 1 and 5,820 in wave 2), corresponding to the number of person-year observations in our main results Table 3.

Fig 1 indicates the number of observations in our main sample per NUTS-2 region and maps mean values of our main dependent and independent variable across NUTS-2 regions (NUTS-2 corresponds to a subdivision of the 16 federal states into 38 regions). Respondents in our sample are distributed over all NUTS-2 regions, with 43 in the region with the lowest number of responses (Mean: 242; Median: 183; SD: 137). In Table 2, we report the descriptive statistics on all utilized variables. Panel A) refers to our dependent variable *compliance index*. With 87 percent of measures followed in 2020 and 88 percent in 2021, compliance is stable on average over time. However, there are considerable variation across the items. For example, avoiding contact with people at particular risk and avoiding public transportation decreased by 7 and 6 percentage points, respectively. In contrast, the use of masks in everyday life has risen from just under two-thirds to 97 percent. This reflects the nationwide obligation to wear a mask in public only since April 29, while approximately two-thirds of the interviews were conducted before that date. Individuals complied most strongly with the measures of regularly washing their hands and with avoiding touching other people. Overall, mean compliance is higher in the west compared to the northeastern (former GDR) parts of Germany (left map in Fig 1). Panel B) of Table 2 reports the descriptives of our main independent variable *satisfaction with the government’s COVID-19 crisis management* and the right map in Fig 1 plots its mean value across NUTS-2 regions. The mean value of satisfaction with the management of the COVID-19 crisis was 6.8 in 2020 compared to 5.8 in 2021 (on the scale from 0 low—10 high). Similarly to compliance, satisfaction is lower in the eastern part of Germany compared to the west. To the naked eye, the maps may suggest a positive correlation between compliance and satisfaction at the NUTS-2 region level, but at 23% it is only weak and not statistically significant.

Panels C) and D) show the descriptive statistics of our instrumental variables. Panel C) shows individuals’ political party preferences before the outbreak of the pandemic. Among respondents surveyed in 2020, more than 50% did not lean toward any political party, approximately 15% leaned toward the conservative party (the CDU resp. CSU in Bavaria), and 10% leaned toward the Social-Democratic party (SPD). The extreme parties on the left (Die Linke) and right (AfD) of the political spectrum were favored by 4% and 3%, respectively. These shares of pre-pandemic party preferences remain nearly unchanged among 2021 respondents. Panel D) shows individuals’ leisure time activities before the pandemic. In 2020, 23% said that they used social media but did not read newspapers daily or at least once per week, 19% read only newspapers, and 53% said that they used both types of media. Also, these proportions remain nearly unchanged when comparing to individuals interviewed in 2021.

Panel E) shows summary statistics for the individual control variables. At just under 55 years of age, respondents in 2021 are about a year older than in 2020. Around 60% of respondents are female and about one third of respondents lives together with children in their household. While only around 8% have at the most lower secondary education (ISCED-2011: 0, 1, 2), slightly more than half have at the most post-secondary, non-tertiary education (ISCED-2011: 3, 4), almost a quarter at the most a Bachelor (ISCED-2011: 5, 6) and 15% a Master or Doctoral degree (ISCED-2011: 7, 8). More than 80% of respondents have no migration background, i.e. they were born in Germany and neither of the parents was born abroad. Respondents live in houses and apartments where one household member has an average of 51–52 square meters of living space. They show a mean satisfaction with their own health of 6.7 on the scale from 0 (low)—10 (high). Almost one quarter has been working in home-office at least partly and up to 10% have been in short-time work during the COVID-19 pandemic. About 43% of respondents were not employed just before the COVID-19 pandemic and the majority of employed individuals was in occupations whose main task can—according to the so-called task-based approach literature [37, 38]—be classified as “analytical non-routine” (22% of all respondents), followed by cognitive routine, manual non-routine, interactive non-routine and manual routine tasks. Controlling for the main occupational task prevents omitted variable bias that might arise from the task affecting both satisfaction and compliance, e.g. because employees who have frequent interactions with customers have difficulty protecting themselves from exposure to the virus and are dissatisfied with the enforcement of the rules in place.

The average time between the mean interview dates in the 2020 (calendar week 17) and 2021 (calendar week 4) survey is 40 weeks. During this period, satisfaction with the government’s COVID-19 crisis management decreased by about 1 point on the 0–10 scale. These results are in line with other surveys in Germany that show high overall satisfaction with government management in the early phase of the pandemic [39]. In an international comparison, German residents display only a modest level of satisfaction with the imposed health measures to mitigate the pandemic. Using survey data for Austria in the period from April 17 to 29 2020, [40] report a mean level of satisfaction of 1.34—on a scale from -3 (dissatisfied) to +3 (satisfied). Using a Chinese online survey of 20,000 respondents at the end of April 2020, [41] report a value of 39.2—on a scale from 10 to 50. Compared to these findings, our results reveal a somewhat lower mean value of satisfaction. In contrast, using survey data for the UK collected at the end of April 2020, [42] reports that 51% of Britons approved government’s handling of the COVID-19 situation, while 30% disapproved it.

Looking at the summary statistics of regional control variables in panel F), the mean unemployment rate (5.4%) and population density (about 1,100 per square kilometer) in respondents’ municipality around the time of the outbreak of the pandemic is rather stable between the 2020 and 2021 survey. Comparing regional health system capacity in the district between 2020 and 2021 respondents, hospital beds for acute care patients per 1,000 residents only decreased by 0.5% to a level of 6,149. The increase in average COVID-19 incidence rate at the county level from 16 to 107 between the time of the 2020 and 2021 surveys reflects the overall increase in infection rates during this period.

### 3.2 Multivariate correlations analysis

In Table 3, columns (1)−(3) (sample of 2020) and (5)−(7) (sample of 2021) show the OLS results of Eq (1) (coefficients and standard errors are multiplied with 100 for presentation). Column (1) and (5) include parsimonious sociodemographic controls such as gender, age, age squared, presence of children in the household, education, migration background and time dummies *δ*_**t**_. The results for our main coefficient of interest *β*_1_ reveal that the compliance index increases significantly with satisfaction. Specifically, compliance increases on average by 1.3 percentage points if an individual’s satisfaction with the government’s COVID-19 crisis management increases—everything else constant—by 1 point on the 0-to-10 scale. Effect sizes are remarkably similar between the 2020 and 2021 sample and remain virtually unchanged when we add further individual control variables such as being employed and type of occupation before the beginning of the COVID-19 pandemic, dummy variables for being in short-time work and working in home-office during the COVID-19 pandemic (columns 2 and 5) as well as district and municipality control variables and NUTS2-level fixed effects in columns (3) and (7).

**Table 3:** Compliance and satisfaction with crisis management, OLS & 2SLS regressions.

Outcome: Compliance with COVID-19 protection measures, 9-item index	Sample: SOEP-CoV, wave 1 participants (2020)				Sample: SOEP-CoV, wave 2 participants (2021)			
	(1) OLS	(2) OLS	(3) OLS	(4) 2SLS	(5) OLS	(6) OLS	(7) OLS	(8) 2SLS
Satisfaction with Covid-19 crisis management of German federal government (0 low—10 high)	1.311***	1.256***	1.263***	3.265***	1.242***	1.219***	1.183***	1.621***
	(0.130)	(0.130)	(0.130)	(0.762)	(0.118)	(0.117)	(0.113)	(0.452)
Strength of political party preference	0.029	-0.007	0.050	-0.190	0.035	0.002	0.009	-0.065
(1 No or very weak—5 very strong)	(0.190)	(0.189)	(0.188)	(0.234)	(0.178)	(0.178)	(0.177)	(0.198)
Female	2.851***	2.784***	2.690***	2.584***	0.098	0.271	0.455	0.336
	(0.740)	(0.747)	(0.727)	(0.716)	(0.553)	(0.563)	(0.544)	(0.546)
Child in household	-0.595	-0.848	-0.896	-0.943	-0.244	-0.251	-0.610	-0.707
	(1.076)	(1.090)	(1.071)	(1.029)	(1.020)	(1.026)	(0.963)	(0.963)
Female × Child in household	-0.920	-0.648	-0.617	-0.555	0.943	0.855	0.770	0.815
	(1.164)	(1.186)	(1.191)	(1.160)	(1.015)	(1.003)	(0.995)	(0.983)
Age	0.068	0.094	0.087	0.073	0.005	0.071	0.060	0.062
	(0.090)	(0.093)	(0.095)	(0.097)	(0.093)	(0.099)	(0.097)	(0.096)
Age, squared	-0.001	-0.001	-0.001	-0.001	0.000	-0.000	-0.000	-0.000
	(0.001)	(0.001)	(0.001)	(0.001)	(0.001)	(0.001)	(0.001)	(0.001)
Highest education: Post-secondary non-tertiary	0.298	0.265	0.310	-0.678	0.755	0.852	0.922	0.725
(ref: lower secondary)	(0.964)	(0.971)	(0.972)	(1.076)	(0.913)	(0.899)	(0.845)	(0.865)
Bachelor	0.255	-0.398	-0.208	-1.954	1.142	0.943	1.591*	1.228
	(1.029)	(1.048)	(1.073)	(1.255)	(1.010)	(1.008)	(0.945)	(1.002)
Master or Doctoral	1.236	0.211	0.521	-1.364	1.211	0.669	1.137	0.726
	(1.028)	(1.049)	(1.070)	(1.249)	(1.181)	(1.164)	(1.053)	(1.117)
Direct migration background (ref: no)	-3.049***	-2.955***	-2.729***	-2.729***	-2.395***	-2.349***	-2.764***	-2.792***
	(0.797)	(0.788)	(0.830)	(0.868)	(0.787)	(0.790)	(0.767)	(0.777)
Indirect migration background	0.870	0.846	1.170	0.995	1.210	1.228	1.268	1.372*
	(0.885)	(0.907)	(0.948)	(1.060)	(0.922)	(0.904)	(0.847)	(0.830)
Living space per person in household (sqm)	-0.008	-0.008	-0.010	-0.010	0.000	-0.001	-0.010	-0.011
	(0.009)	(0.009)	(0.009)	(0.009)	(0.009)	(0.009)	(0.008)	(0.008)
Health satisfaction, measured before	-0.245**	-0.224*	-0.238*	-0.496***	-0.149	-0.106	-0.078	-0.137
Covid-19 pandemic (0 low—10 high)	(0.119)	(0.120)	(0.122)	(0.167)	(0.107)	(0.109)	(0.106)	(0.121)
Has at least been partly in home-office during Covid-19 pandemic		1.977***	2.144***	1.369		1.774***	1.793***	1.596**
		(0.745)	(0.749)	(0.910)		(0.608)	(0.611)	(0.652)
Has been in short-time work during Covid-19 pandemic		1.499*	1.319	1.315		0.682	1.457	1.406
		(0.825)	(0.838)	(0.894)		(0.953)	(0.945)	(0.936)
Occupation before Covid-19 pandemic								
(ref: analytical non-routine)								
Interactive non-routine tasks		-1.443*	-1.348*	-1.371		-0.993	-0.938	-0.933
		(0.798)	(0.803)	(0.875)		(0.867)	(0.857)	(0.851)
Cognitive routine tasks		-1.831**	-1.818**	-1.791*		-0.396	-0.331	-0.322
		(0.888)	(0.893)	(0.918)		(0.752)	(0.750)	(0.743)
Manual routine tasks		-3.686**	-3.493**	-3.051*		1.482	1.528	1.621
		(1.621)	(1.634)	(1.725)		(1.266)	(1.219)	(1.211)
Manual non-routine tasks		-2.239**	-2.185**	-1.247		-2.311**	-2.165**	-2.131**
		(0.996)	(0.998)	(1.098)		(0.979)	(0.944)	(0.941)
Not employed		0.205	0.445	0.259		2.049**	2.293***	2.249***
		(0.755)	(0.770)	(0.817)		(0.794)	(0.764)	(0.753)
Unemployment rate in municipality, March-31–2020			-0.105	-0.085			0.218	0.227
			(0.142)	(0.144)			(0.183)	(0.182)
Log population density in municipality, March-31–2020			0.205	0.131			-0.230	-0.223
			(0.280)	(0.293)			(0.322)	(0.320)
Hospital beds in district per 1,000 inhabitants, 2016			-0.190**	-0.217**			-0.133	-0.141
			(0.089)	(0.096)			(0.116)	(0.115)
Covid-19 incidence rate in district			0.001	-0.004			-0.014*	-0.014*
(last 7 days before interview, per 100k)			(0.017)	(0.018)			(0.008)	(0.008)
NUTS2 region of residence fixed effects	No	No	Yes	Yes	No	No	Yes	Yes
Interview calender-week fixed effects	Yes	Yes	Yes	Yes	Yes	Yes	Yes	Yes
Observations	3,390	3,390	3,390	3,390	5,820	5,820	5,820	5,820
R2 adjusted	0.064	0.072	0.072		0.039	0.044	0.078	
Underidentification:								
Kleibergen-Paap rk LM statistic				52.495				87.661
p-value Kleibergen-Paap rk LM				0.000				0.000
Weak identification:								
Kleibergen-Paap rk Wald F statistic				17.226				44.850
Overidentification: Hansen J statistic				9.644				8.801
p-value of Hansen J				0.140				0.185
Dependent variable mean	0.873	0.873	0.873	0.873	0.879	0.879	0.879	0.879

Source: SOEP-CoV waves 1, 2 and SOEP-Core of Socio-Economic Panel (SOEP) (v37). Standard errors clustered at district-level in parentheses. Coefficients and SEs multiplied with 100 for presentation.

* p < 0:10,

** p < 0:05,

*** p < 0:01.

Excluded instruments: 7 dummy variables for political party preference. All models control for a regression constant and missing values in control variables.

These results show a strong positive and significant correlation between subjective satisfaction and protective behavior. However, they may be biased in presence of endogeneity issues, as discussed in section 2.4. Therefore, we apply an instrumental variable (IV) approach to address potential endogeneity issues.

### 3.3 Instrumental variable approach

In Table 3, columns (4) and (8) show the results of our main IV estimates. In 2020, the coefficient on satisfaction with the government’s COVID-19 crisis management almost triples compared to those from the OLS estimates. In 2021, it is almost 40% larger than the OLS coefficient. These results confirm that endogeneity biases the measured effects downward in the simple correlation analysis. An increase in satisfaction by one unit on the 11-point scale raises individuals’ compliance by 3.3 percentage points in 2020 and 1.6 in 2021. Put differently, if the overall average satisfaction with the government’s COVID-19 crisis management were at the average level for AfD supporters (4.4 in 2020 and 3.1 in 2021 on the 0–10 scale) (i.e., if it were to decline by about 2.5 points), the compliance rate would drop by 4 to 8 percentage points. Overall, the results highlight an important implication. At the time of the survey, public perceptions of the acute health risk of the SARS-CoV-2 virus were quite high. Thus, the effect size is large given the exponential rise in the number of infection cases in Germany and many other countries around the world in the survey phase.

Table 3 reveals further findings. Compliance of women is significantly more pronounced in 2020 than that of men—on average almost 3 percentage points. One reason why this difference is no longer apparent in 2021 could be that some protection measures were made legally mandatory over the course of the pandemic. Furthermore, individuals with a direct migration background comply less than those without migration background in both surveys. Individuals who report higher satisfaction with their own health prior to the pandemic comply less in the 2020 survey.

## 4 Limitations and robustness checks

The results presented so far have some limitations, which we first present briefly before discussing each limitation in detail. We also present robustness checks to assess the sensitivity of our main results to each limitation. First, since our compliance index combines several compliance dimensions, our results do not take into account potential heterogeneity in the effect of satisfaction on different compliance dimensions. Second, our instrumental variable estimates rely on strong assumptions and are only valid if these assumptions are met. Third, the linear OLS and IV estimators assume an unbounded outcome variables although the compliance index is bounded to the [0; 1] interval. Fourth, our regression analysis does not account for potential non-random participation of respondents in the probability-based survey. Finally, it is unclear to what extent our results can be generalised beyond the German setting given the different health policies introduced over the study period.

### 4.1 Single compliance components

In Table A.2 in S1 Appendix, we disaggregate our compliance index to estimate the effect of satisfaction with the government’s COVID-19 crisis management on each compliance dimension separately. We apply—similar to our preferred model in Table 3, cols. 4, 8—2SLS with political party preferences as instrumental variables. Again, note that coefficients and standard errors are multiplied with 100 for presentation. Overall, effects are more often positive and statistically significant in 2020 than in 2021 (2020: 7 out of 9; 2021: 4 out of 9) and effect sizes are larger. This could be due to some compliance measures having become mandatory over the course of the pandemic, and to increased knowledge about their effectiveness as well as about the health risks in the event of an infection.

Higher satisfaction decreased contact with at-risk groups, shopping at peak hours, being in crowds of people, handshaking or hugging both in 2020 and 2021. It only led to maintaining a larger distance from symptomatic people, more thorough hand washing and more frequent wearing of face masks in 2020. No effects are visible on avoidance of public transport as well as trips, likely due to non-deferrability.

### 4.2 Alternative set of instrumental variables

In this section, we present results for an alternative set of instrumental variables as a robustness check. Specifically, we exploit variation in how frequently respondents used social media and read newspapers before the COVID-19 pandemic. This information was elicited in the panel survey wave in 2019 (see section 2.2). Columns 5−8 of Table A.1 in S1 Appendix show that these instruments are highly relevant in the first-stage. People who only use social media at least one per week (but not read newspapers at least once per week) perceive the government’s COVID-19 crisis management significantly worse than those who use both types of media at least weekly. We suspect that conspiracy theories, which are often spread on social networks such as Facebook or Twitter, are important here [43]. One potential interpretation could be that individuals who intensively use social media are less informed about the numbers of infection cases and the health-protective measures.

Table A.3 in S1 Appendix shows the second-stage 2SLS results. We control for the same set of sociodemographic and regional control variables as in our main specification (columns 4 and 8 of Table 3). The results confirm that satisfaction with the government’s COVID-19 crisis management has a positive and statistically significant effect on compliance. A rise in satisfaction by one unit induces higher compliance with health-protective measures by 3.9 (2.3) percentage points in 2020 (2021). When we compare the coefficients with those of columns (4) and (8) in Table 3, both confidence intervals overlap.

### 4.3 Non-linear estimator: Probit-IV

Our main dependent variable is the compliance index, which is defined as the share of adhered measures out of 9 options. By definition, the share is bounded to the [0;1] interval. Although many studies have used the linear estimator to identify effects of treatment variables on fractional outcomes [44], the linear OLS and IV estimators assume an unbounded outcome. Thus, applying the linear estimator to a fractional outcome may miss important non-linearities and produce biased parameter estimates as well as incorrect confidence intervals, e.g. ranging beyond the 1–0 thresholds [45]. This problem becomes more severe, the closer the mean of the outcome variable—and the predicted value—is to the interval thresholds. In such cases, the assumption of normally distributed residuals may not be met. Given that the mean value of our main dependent variable (*compliance index*) is quite high at almost 0.9, we perform non-linear regressions to rule out such concerns. Unfortunately, there is no ready-to-use estimator for fractional outcomes implemented in the commonly used statistical software packages. Therefore, we transform our compliance index into a dummy variable. Specifically, we code the dummy to 1 if respondents adhere to all out of 9 compliance measures and 0 if they adhere to 8 or less (the median compliance is 8 out of 9). Moreover, we replicate the results from Table A.2 in S1 Appendix, running 9 separate regressions for each component of our compliance index.

Results are shown in Table A.4 in S1 Appendix. Similar to our main results, satisfaction with the government’s crisis management positively affects compliance and effects are stronger in the 2020 survey. While the probability to adhere to all out of 9 compliance measures increases, on average, by more than 4 percentage points in 2020 if satisfaction increases by 1 on the 11-point scale, the effect reduces to 2.5 percentage points in 2021 (models 1 and 11). The component-wise results are close to the linear 2SLS IV estimator in Table A.2 in S1 Appendix.: although effect sizes are marginally smaller in the non-linear case, they are positive and statistically significant across the same compliance components and survey years. Only for *regularly washing hands (at least 20 seconds with soap and water)*, which was weakly statistically significant in the linear specification, we do not get significant results in the non-linear case. Also notable is *wearing a protective mask when running errands or on public transport* for which the coefficient in the 2020 non-linear model more than triples compared to the 2020 linear one (9.3 vs. 2.9). The reason for this is the larger variation compared to the other compliance components: in the 2020 survey, only 63% of respondents reported wearing a mask (other components more than 80%). The larger effect size results from the higher slope of the link function of the probit estimator in the middle range compared to the linear model.

### 4.4 Applying survey weights

Although each tranche of SOEP-CoV was structured such that household composition within each tranch was representative of all private households in Germany [25], one may be concerned about non-random participation of respondents in the survey. This would induce bias in our estimates if, for example, individuals that are dissatisfied with the government’s crisis management and at the same time comply strongly are systematically underrepresented in the survey. In order to rule out this mechanism, we re-estimate the effect of satisfaction with the government’s COVID-19 crisis management on compliance applying survey weights. The calculation of weights builds on the existing household weights from participation in the pre-pandemic SOEP survey. These were, first, adjusted for successive dropouts in participation in SOEP-CoV (based on about 400 characteristics from previous participation in the panel survey, regional variables at the county level of residence, and telephone contact histories). Second, they were marginally adjusted according to various population distributions from the German micro-census (such as distribution of household number and size by state). Detailed information on the generation of the weights can be found in [46].

In column 1 of Table A.5 in S1 Appendix, we replicate our main result from Table 3 (col. 4) for the 2020 (wave 1) sample, applying the weights. The coefficient is slightly larger (not statistically significant). Note that the estimation sample is restricted to tranches 2–4 (out of 9) of the wave 1 sample due to changing questionnaire composition (see section 2.1). Specifically, *wearing a protective mask when running errands or on public transport* was not asked to respondents of tranche 1 in 2020, which makes up about one quarter of responses in SOEP-CoV wave 1. In order to increase sample size, in column 2 of Table A.5 in S1 Appendix, we exclude the face mask dimension from the compliance index, which increases our sample size by more than 1,500 individuals. Still respondents of tranches 5–9 cannot be included as they were not asked on *satisfaction with the government’s COVID-19 crisis management* (they make up about 20% of all SOEP-CoV respondents in wave 1). Also in this specification, which does not apply survey weights, the coefficient is very close and statistically equal to our preferred model in Table 3, col. 4). The same holds true for column 3, which differs from column 2 only in the inclusion of weights. Finally, the coefficient for the 2021 (wave 2) sample with survey weights in column 4 of Table A.5 in S1 Appendix is positive and statistically significant and confidence intervals overlap with the coefficient from the corresponding model in Table 3 (col. 8). The 2021 weights explicitly include non-response adjustments that balance varying response probabilities from wave 1 to wave 2, thus, addressing potential attrition bias in our results. Taken together, the exercises in Table A.5 in S1 Appendix do not indicate any biasing effects from potential non-random survey participation.

## 5 Discussion

Using unique panel data, this paper investigates the impact of subjective satisfaction with the German federal government’s management of the COVID-19 crisis on compliance with health-protective measures. To identify the causal effect, we employ an IV approach. In doing so, we exploit the longitudinal nature of the panel sample to overcome the endogeneity concerns. The vast majority of recent survey studies on compliance with public health policies rely on ad hoc (online) surveys conducted during the ongoing pandemic. Notable large-scale and multi-purpose exceptions are the Swiss Household Panel [47], UK Household Longitudinal Study [48] and the Understanding America Study [49], which can be used to analyze changes over the pandemic based on at least one pre-pandemic observation for the same individual (see [50] for a systematic review). This also applies to some surveys with more targeted topics, such as the Swedish Pregnancy Panel [51]. In contrast, the SOEP-CoV data are based on an ongoing, representative German panel study running since 1984 (SOEP-CORE). By utilizing SOEP-CoV data instead of convenience sample data, we minimize potential undercoverage and nonresponse biases. Moreover, combining the SOEP-CoV data with detailed individual and household panel data enables us to draw causal inferences based on the information on individual preferences and attitudes collected during *and* before the COVID-19 crisis. We instrument subjective satisfaction with the government’s COVID-19 crisis management with political party preferences prior to the COVID-19 crisis. The instrument exploits the lower satisfaction with the government’s crisis management of individuals with political preferences for right-wing populist parties (e.g., AfD).

This paper contributes to at least two strands of the literature. First, a new set of studies emerged during the COVID-19 to examine compliance with health measures. One set of studies tests which factors relate to protective behavior during the COVID-19 pandemic [31, 52–55]. Previously, [56] also studied the relationship between preventive health behavior and flu vaccine take-up rates. For example, using online survey data for the U.S., [53] show that during the COVID-19 pandemic, compliance has depended on substantive moral support and social norms. Using survey data for the US, the UK and six member states of the European Union (EU), [54] show that compliance with health measures relates to threat perceptions and feelings of efficacy. [52] use survey data for Italy and show that intentions to comply with self-isolation restrictions depend on the duration of the protective measures and expectations about the extension of such measures. [31] show that partisan bias relates to risk perception and protective behavior. Their results reveal that counties with a higher share of Donald Trump voters are associated with less search activity regarding information on the virus and less engagement in social distancing behavior. [32] offer a discussion on political beliefs, partisan affiliation and compliance with health-protective measures. However, these studies mainly measure social and protective behavior as it has occurred during the pandemic. Since the COVID-19 pandemic caused an unexpected and major disruption in many people’s lives, beliefs, political preferences and social behavior might have changed at the same time. This in turn could induce spurious correlations and limit the informative value of linkages between variables observed during the pandemic. Using an instrumental variable approach, this study takes a further step towards identifying the causal effect of social behavior on compliance with COVID-19 mitigation measures.

Our results show that a one unit increase in subjective satisfaction on the 11-point scale raises the protective behavior index by 2–3 percentage points. The effect size decreases as the pandemic progresses, probably because a number of containment measures became mandatory. Also, our results demonstrate that compliance is strongly related to an individual’s profession and working conditions. The results also hold when we use an alternative IV approach, instrumenting satisfaction with the government’s crisis management with media consumption prior to the COVID-19 crisis. Moreover, these results are almost up to three times larger than those of the simple OLS estimates used in recent studies in the context of COVID-19, indicating a downward bias due to endogeneity and reverse causality issues.

These results augment the existing findings showing that preferences are correlated with contributions to public goods [57, 58], voting behavior [59], and compliance in crisis situations [31, 52, 55, 60, 61]. They also contribute to the general discussion about social preferences in the context of public goods [59, 60, 62–64]. For example, [59] find that subjective well-being explains retrospective voting behavior in favor of the incumbent. Using data for the US and for Germany, [60, 65] show that trust in institutions and social capital, respectively, determine compliance with COVID-19 measures. In this context, [64] find that in democratic societies, people’s confidence in their government’s actions improves more the efficacy of COVID-19 mitigation measures than implementation of strict protective measures. In relation to this literature, we investigate how preferences and attitudes with respect to social action have affected individual responses to policies during the pandemic crisis. Our findings suggest that, through its compliance-enhancing effect, subjective satisfaction with the COVID-19 crisis management increases the effectiveness of relatively mild public health measures. This can potentially increase the cost-effectiveness of anti-pandemic policies by preventing the need for more stringent measures, such as lockdowns with curfews. Hence, our results highlight that satisfaction preference is an important parameter to social planer’s trade-off between economic costs and welfare benefits of uniform policies.

Second, our study is also related to the discussion on happiness, public policy and governance [12, 66–68]. Happiness or subjective well-being is a useful approximation of individual utility [12]. Utility is a term used to rank individual’s preferences over a set of their choices or actions. According to economic theory, individuals choose the option that yields the highest utility less the costs induced by the action. By inducing social action, policy entails costs for some individuals. Policymakers need to take into account individual utility to assess the net effects of policies on society. If individuals are convinced of the benefits of a government’s motives and actions, they are more likely to perceive the utility associated with certain restrictions. This could sway their preferences in favor of collective action and induce them to accept certain individual costs associated with restrictions more readily. [68] shows that political freedom and private freedom correlate strongly with happiness in rich and capable countries. More generally, greater government accountability, effectiveness, and stability is associated with higher subjective well-being [12]. Empirical evidence shows that happier people tend to have pro-democratic and pro-market attitudes [67]. Similarly to this line of research, we address how satisfaction correlates with political attitudes and media consumption and how these in turn affect protective behavior. Satisfaction scores of AfD supporters are, on average, more than 2 points lower than those of the reference group with no party preference (on the 11-point scale). Individuals who predominantly and frequently use social media are approximately 0.5 points less satisfied than those who frequently read newspapers.

## 6 Conclusion

Overall, our findings provide several important insights. First, they highlight that the efficacy of COVID-19 mitigation measures depends, inter alia, on individual preferences for government policy. Second, political attitudes and sources of information seem to play an important role in how a society responds during a pandemic crisis. They shape confidence and trust in institutions and government action. Third, the efficacy of uniform policy measures depends on the costs and benefits of social action. Thus, policymakers need to take into account individual preferences for social action to assess the net effects of uniform policies on society. Finally, our results indicate that the effectiveness of policy measures in various domains, such as the health system, social security or taxation, especially during pandemic crises, need to carefully consider individual preferences for social action and provide targeted information.

## Supporting information

S1 Appendix (PDF)
