# Supplementary material for: Satisfaction with pandemic management and compliance with public health measures: Evidence from a German household survey on the COVID-19 crisis
Source: PLoS One. 2023 Feb 21;18(2):e0281893. doi: 10.1371/journal.pone.0281893 (PMC9942998; doi:10.1371/journal.pone.0281893)
Supplement: S1 Appendix — (PDF) [file pone.0281893.s001.pdf]

Online Appendix

Submitted Manuscript: Satisfaction with Pandemic Management and Compliance with Public Health Measures: Evidence from a German Household Survey on the COVID-19 Crisis

Table A.1

Political preferences, media consumption and satisfaction (first-stage IV regressions)

IV, measured before the pandemic:	Political party preferences				Media consumption			
Satisfaction with Covid-19 crisis management of German	Sample: 2020		Sample: 2021		Sample: 2020		Sample: 2021	
federal government (0 low - 10 high)	(1)	(2)	(3)	(4)	(5)	(6)	(7)	(8)
Social-Democrats (SPD) (ref: no party preference)	0.451**	0.389*	0.600***	0.492***				
	(0.208)	(0.208)	(0.167)	(0.165)				
Conservatives (CDU, CSU)	0.775***	0.732***	0.893***	0.813***				
	(0.218)	(0.219)	(0.158)	(0.159)				
Liberals (FDP)	0.051	0.047	-0.073	-0.173				
	(0.298)	(0.295)	(0.248)	(0.247)				
Greens	0.586***	0.520**	0.850***	0.727***				
	(0.221)	(0.218)	(0.164)	(0.162)				
Far-left (Die Linke)	0.138	0.298	-0.029	0.052				
	(0.253)	(0.259)	(0.193)	(0.191)				
Far-right (AFD)	-2.149***	-2.093***	-2.342***	-2.305***				
	(0.370)	(0.376)	(0.270)	(0.271)				
Other	-0.328	-0.318	-0.107	-0.179				
	(0.389)	(0.386)	(0.313)	(0.308)				
Neither used social media, nor read newspapers					-0.322*	-0.273	-0.240	-0.202
before Covid-19 pandemic (ref: both)					(0.194)	(0.197)	(0.150)	(0.152)
only used social media					-0.582***	-0.561***	-0.523***	-0.515***
					(0.111)	(0.110)	(0.090)	(0.090)
only read newspapers					-0.006	0.040	-0.021	0.023
					(0.098)	(0.098)	(0.083)	(0.083)
Strength of political party preference	0.026	0.022	0.043	0.049	0.123***	0.109***	0.182***	0.158***
(1 No or very weak - 5 very strong)	(0.074)	(0.074)	(0.055)	(0.055)	(0.032)	(0.032)	(0.025)	(0.025)
Female	-0.032	-0.018	0.170**	0.190***	0.061	0.070	0.265***	0.284***
	(0.090)	(0.091)	(0.071)	(0.071)	(0.093)	(0.094)	(0.072)	(0.072)
Child in household	0.001	0.003	0.154	0.138	0.031	0.020	0.240**	0.209**
	(0.138)	(0.139)	(0.108)	(0.107)	(0.139)	(0.140)	(0.106)	(0.105)
Female $\times$ Child in household	-0.032	-0.039	-0.060	-0.068	-0.016	-0.016	-0.083	-0.094
	(0.146)	(0.148)	(0.132)	(0.132)	(0.149)	(0.151)	(0.130)	(0.130)
Age	0.009	0.014	-0.000	0.003	-0.008	-0.003	-0.019	-0.015
	(0.014)	(0.014)	(0.012)	(0.012)	(0.014)	(0.014)	(0.013)	(0.013)
Age, sq.	0.000	0.000	0.000	0.000	0.000	0.000	0.000**	0.000**
	(0.000)	(0.000)	(0.000)	(0.000)	(0.000)	(0.000)	(0.000)	(0.000)
Highest education: Post-secondary non-tertiary	0.477***	0.499***	0.365***	0.428***	0.406**	0.444***	0.334**	0.414***
(ref: Lower secondary)	(0.162)	(0.162)	(0.133)	(0.132)	(0.159)	(0.159)	(0.135)	(0.134)
Bachelor	0.762***	0.814***	0.636***	0.747***	0.730***	0.802***	0.629***	0.767***
	(0.169)	(0.171)	(0.147)	(0.146)	(0.169)	(0.171)	(0.150)	(0.148)
Master or Doctoral	0.834***	0.852***	0.755***	0.835***	0.793***	0.826***	0.752***	0.854***
	(0.183)	(0.190)	(0.147)	(0.147)	(0.184)	(0.192)	(0.151)	(0.150)
Direct migration background (ref: no)	0.106	0.020	0.261**	0.124	0.105	0.005	0.242**	0.082
	(0.118)	(0.119)	(0.115)	(0.115)	(0.123)	(0.125)	(0.120)	(0.119)
Indirect migration background	0.159	0.073	-0.104	-0.192	0.173	0.070	-0.133	-0.249*
	(0.189)	(0.188)	(0.127)	(0.127)	(0.184)	(0.184)	(0.134)	(0.133)
Living space per person in household (sqm)	0.000	-0.000	0.002*	0.001	0.001	0.000	0.003**	0.001
	(0.001)	(0.001)	(0.001)	(0.001)	(0.001)	(0.001)	(0.001)	(0.001)
Health satisfaction, measured before Covid-19 pandemic	0.126***	0.122***	0.129***	0.125***	0.128***	0.123***	0.135***	0.129***
(0 low - 10 high)	(0.018)	(0.018)	(0.015)	(0.015)	(0.018)	(0.018)	(0.015)	(0.015)
Has at least been partly in home-office	0.393***	0.372***	0.439***	0.411***	0.413***	0.378***	0.474***	0.437***
during Covid-19 pandemic	(0.111)	(0.114)	(0.080)	(0.082)	(0.115)	(0.118)	(0.081)	(0.082)
Has been in short-time work during Covid-19 pandemic	0.033	0.016	0.092	0.098	0.030	0.007	0.122	0.137
	(0.135)	(0.136)	(0.134)	(0.134)	(0.137)	(0.139)	(0.139)	(0.138)
Occupation before Covid-19 pandemic (ref: analytical non-routine)								
Interactive non-routine tasks	-0.016	-0.039	-0.027	-0.041	0.077	0.049	0.036	0.019
	(0.129)	(0.131)	(0.120)	(0.119)	(0.134)	(0.135)	(0.123)	(0.122)
Cognitive routine tasks	-0.057	-0.043	-0.056	-0.029	-0.025	-0.009	-0.059	-0.026
	(0.125)	(0.125)	(0.097)	(0.098)	(0.128)	(0.129)	(0.100)	(0.100)
Manual routine tasks	-0.134	-0.145	-0.175	-0.133	-0.228	-0.234	-0.250	-0.202
	(0.234)	(0.233)	(0.193)	(0.190)	(0.246)	(0.245)	(0.197)	(0.194)
Manual non-routine tasks	-0.420***	-0.447***	-0.018	-0.004	-0.419**	-0.442***	-0.080	-0.059
	(0.157)	(0.158)	(0.119)	(0.121)	(0.164)	(0.165)	(0.122)	(0.123)
Not employed	0.135	0.141	0.092	0.127	0.099	0.100	0.052	0.102
	(0.137)	(0.137)	(0.115)	(0.116)	(0.138)	(0.138)	(0.114)	(0.115)
Unemployment rate in municipality, March-31-2020		-0.004		-0.012		-0.012		-0.020
		(0.022)		(0.020)		(0.023)		(0.020)
Log population density in municipality, March-31-2020		0.038		-0.006		0.041		-0.014
		(0.041)		(0.033)		(0.042)		(0.033)
Hospital beds in district per 1,000 inhabitants, 2016		0.012		0.016		0.013		0.019*
		(0.014)		(0.010)		(0.014)		(0.010)
Covid-19 incidence rate in district		0.002		-0.001		0.002		-0.001
(last 7 days before interview, per 100k)		(0.002)		(0.001)		(0.002)		(0.001)
NUTS2 region of residence fixed effects	No	Yes	No	Yes	No	Yes	No	Yes
Interview calender-week fixed effects	Yes	Yes	Yes	Yes	Yes	Yes	Yes	Yes
Observations	3,390	3,390	5,820	5,820	3,390	3,390	5,820	5,820
R2 adjusted	0.125	0.130	0.124	0.132	0.091	0.100	0.081	0.095
Dependent variable mean	6.815	6.815	5.797	5.797	6.815	6.815	5.797	5.797

Source: SOEP-CoV waves 1, 2 and SOEP-Core of Socio-Economic Panel (SOEP) (v37).

Standard errors clustered at district-level in parentheses. * $p < 0.10$, ** $p < 0.05$, *** $p < 0.01$. All models control for a regression constant and missing values in control variables.

Table A.2
Compliance components, 2SLS estimator (IV: party preference)

Compliance measure (dependent variable):	Contact	Transport	Trips	Shopping	Crowds	Symptom	Touch	Wash	Masks
	**Sample: SOEP-CoV, wave 1 participants (2020)**								
	(1)	(2)	(3)	(4)	(5)	(6)	(7)	(8)	(9)
Satisfaction with Covid-19 crisis management of German	5.257***	1.562	1.339	5.560***	3.851***	3.736***	2.548**	2.323*	2.903*
federal government (0 low - 10 high)	(1.683)	(1.444)	(1.151)	(1.676)	(1.122)	(1.316)	(1.050)	(1.239)	(1.626)
Same control variables as in main results (Table 3, cols. 4, 8)	Yes	Yes	Yes	Yes	Yes	Yes	Yes	Yes	Yes
Observations	3,390	3,390	3,390	3,390	3,390	3,390	3,390	3,388	3,390
Underidentification: Kleibergen-Paap rk LM statistic	52.495	52.495	52.495	52.495	52.495	52.495	52.495	51.710	52.495
p-value Kleibergen-Paap rk LM	0.000	0.000	0.000	0.000	0.000	0.000	0.000	0.000	0.000
Weak identification: Kleibergen-Paap rk Wald F statistic	17.226	17.226	17.226	17.226	17.226	17.226	17.226	16.872	17.226
Overidentification: Hansen J statistic	2.891	10.023	4.274	5.158	19.302	2.657	3.794	15.725	12.152
p-value of Hansen J	0.822	0.124	0.640	0.524	0.004	0.850	0.705	0.015	0.059
Dependent variable mean	0.810	0.847	0.925	0.837	0.956	0.919	0.957	0.976	0.632
	**Sample: SOEP-CoV, wave 2 participants (2021)**								
	(10)	(11)	(12)	(13)	(14)	(15)	(16)	(17)	(18)
Satisfaction with Covid-19 crisis management of German	4.325***	0.389	0.541	3.121***	2.308***	0.649	3.050***	-0.170	0.380
federal government (0 low - 10 high)	(1.204)	(1.054)	(0.877)	(1.063)	(0.814)	(0.992)	(0.821)	(0.500)	(0.506)
Same control variables as in main results (Table 3, cols. 4, 8)	Yes	Yes	Yes	Yes	Yes	Yes	Yes	Yes	Yes
Observations	5,820	5,820	5,820	5,820	5,820	5,820	5,820	5,820	5,820
Underidentification: Kleibergen-Paap rk LM statistic	87.661	87.661	87.661	87.661	87.661	87.661	87.661	87.661	87.661
p-value Kleibergen-Paap rk LM	0.000	0.000	0.000	0.000	0.000	0.000	0.000	0.000	0.000
Weak identification: Kleibergen-Paap rk Wald F statistic	44.850	44.850	44.850	44.850	44.850	44.850	44.850	44.850	44.850
Overidentification: Hansen J statistic	3.677	8.728	3.986	2.052	8.087	13.290	12.750	4.741	3.615
p-value of Hansen J	0.720	0.189	0.679	0.915	0.232	0.039	0.047	0.577	0.729
Dependent variable mean	0.744	0.789	0.878	0.797	0.931	0.911	0.941	0.952	0.968

Source: SOEP-CoV waves 1, 2 and SOEP-Core of Socio-Economic Panel (SOEP) (v37).
Standard errors clustered at district-level in parentheses. Coefficients and SEs multiplied with 100 for presentation. * $p < 0.10$, ** $p < 0.05$, *** $p < 0.01$.
Excluded instruments: 7 dummy variables for political party preference. All models control for the same set of control variables as in our main results (Table 3, cols. 4, 8) of the manuscript, a regression constant and missing values in control variables.
Dependent variables are dummies for: (1) Avoiding contact with at-risk groups (elderly individuals, chronically ill individuals, (2) avoiding public transport, (3) avoiding trips, (4) avoiding shopping at peak hours, (5) staying away from crowds, (6) keeping a distance from symptomatic people, (7) avoiding touching, such as handshaking or hugging, (8) regularly washing hands, (9) wearing masks.

Table A.3

Compliance and satisfaction with crisis management, OLS & 2SLS regressions (IV: media consumption)

Sample:	Wave 1, 2020	Wave 2, 2021
Outcome: Compliance with Covid-19 protection measures, 9-item index	(1) 2SLS	(2) 2SLS
Satisfaction with Covid-19 crisis management of German	3.893***	2.285**
federal government (0 low - 10 high)	(1.287)	(0.999)
Strength of political party preference	-0.266	-0.176
(1 No or very weak - 5 very strong)	(0.265)	(0.250)
Female	2.550***	0.155
	(0.715)	(0.599)
Child in household	-0.958	-0.853
	(1.033)	(0.965)
Female × Child in household	-0.535	0.884
	(1.170)	(0.973)
Age	0.069	0.066
	(0.101)	(0.097)
Age, sq.	-0.001	-0.001
	(0.001)	(0.001)
Highest education: Post-secondary non-tertiary	-0.988	0.425
(ref: Lower secondary)	(1.207)	(0.952)
Bachelor	-2.502	0.676
	(1.586)	(1.271)
Master or Doctoral	-1.956	0.103
	(1.604)	(1.380)
Direct migration background (ref: no)	-2.728***	-2.835***
	(0.897)	(0.794)
Indirect migration background	0.940	1.529*
	(1.112)	(0.841)
Living space per person in household (sqm)	-0.011	-0.011
	(0.009)	(0.009)
Health satisfaction, measured before Covid-19 pandemic	-0.577***	-0.226
(0 low - 10 high)	(0.218)	(0.171)
Has at least been partly in home-office	1.126	1.297*
during Covid-19 pandemic	(1.059)	(0.737)
Has been in short-time work during Covid-19 pandemic	1.314	1.327
	(0.931)	(0.956)
Occupation before Covid-19 pandemic (ref: analytical non-routine)		
Interactive non-routine tasks	-1.378	-0.925
	(0.917)	(0.860)
Cognitive routine tasks	-1.782*	-0.309
	(0.941)	(0.747)
Manual routine tasks	-2.912	1.761
	(1.844)	(1.249)
Manual non-routine tasks	-0.953	-2.080**
	(1.222)	(0.951)
Not employed	0.201	2.182***
	(0.863)	(0.757)
Unemployment rate in municipality, March-31-2020	-0.079	0.241
	(0.147)	(0.184)
Log population density in municipality, March-31-2020	0.107	-0.211
	(0.300)	(0.318)
Hospital beds in district per 1,000 inhabitants, 2016	-0.225**	-0.153
	(0.101)	(0.118)
Covid-19 incidence rate in district	-0.006	-0.014*
(last 7 days before interview, per 100k)	(0.019)	(0.008)
NUTS2 region of residence fixed effects	Yes	Yes
Interview calender-week fixed effects	Yes	Yes
Observations	3,390	5,820
Underidentification: Kleibergen-Paap rk LM statistic	26.220	31.813
p-value Kleibergen-Paap rk LM	0.000	0.000
Weak identification: Kleibergen-Paap rk Wald F statistic	9.686	11.345
Overidentification: Hansen J statistic	7.841	9.546
p-value of Hansen J	0.020	0.008
Dependent variable mean	0.873	0.879

Source: SOEP-CoV waves 1, 2 and SOEP-Core of Socio-Economic Panel (SOEP) (v37).
Standard errors clustered at district-level in parentheses. Coefficients and SEs multiplied with 100 for presentation. * $p < 0.10$, ** $p < 0.05$, *** $p < 0.01$.
Excluded instruments: 3 dummy variables for combinations of (i) frequency of using social media and (ii) frequency of reading newspapers before the Covid-19 pandemic (at least weekly vs. less than weekly). All models control for a regression constant and missing values in control variables.

Table A.4

Robustness with non-linear estimator (Probit-IV)

Compliance measure (dependent variable):	All measures (out of 9)	Single compliance components								
		Contact	Transport	Trips	Shopping	Crowds	Symptom	Touch	Wash	Masks
**Sample: SOEP-CoV, wave 1 participants (2020)**										
	(1)	(2)	(3)	(4)	(5)	(6)	(7)	(8)	(9)	(10)
Satisfaction with Covid-19 crisis management of German	4.175**	4.176***	1.921	1.196	4.510***	2.964***	2.961***	1.681**	1.962	9.263***
federal government (0 low - 10 high)	(1.864)	(1.215)	(1.576)	(0.970)	(1.148)	(1.032)	(0.989)	(0.788)	(1.459)	(2.956)
Same control variables as in main results (Table 3, cols. 4, 8)	Yes	Yes	Yes	Yes	Yes	Yes	Yes	Yes	Yes	Yes
Observations	3,390	3,390	3,390	3,390	3,390	3,390	3,390	3,390	3,388	3,390
Dependent variable mean	0.356	0.810	0.847	0.925	0.837	0.956	0.919	0.957	0.976	0.632
**Sample: SOEP-CoV, wave 2 participants (2021)**										
	(11)	(12)	(13)	(14)	(15)	(16)	(17)	(18)	(19)	(20)
Satisfaction with Covid-19 crisis management of German	2.573**	3.973***	0.318	0.591	2.921***	1.945***	0.644	2.428***	-0.064	0.415
federal government (0 low - 10 high)	(1.230)	(0.952)	(1.144)	(0.813)	(0.935)	(0.683)	(0.961)	(0.655)	(0.499)	(0.437)
Same control variables as in main results (Table 3, cols. 4, 8)	Yes	Yes	Yes	Yes	Yes	Yes	Yes	Yes	Yes	Yes
Observations	5,820	5,820	5,820	5,820	5,820	5,820	5,820	5,820	5,820	5,820
Dependent variable mean	0.463	0.744	0.789	0.878	0.797	0.931	0.911	0.941	0.952	0.968

Source: SOEP-CoV waves 1, 2 and SOEP-Core of Socio-Economic Panel (SOEP) (v37).

Standard errors clustered at district-level in parentheses. Coefficients and SEs multiplied with 100 for presentation. * $p < 0.10$, ** $p < 0.05$, *** $p < 0.01$.

Excluded instruments: 7 dummy variables for political party preference. All models control for the same set of control variables as in our main results (Table 3, cols. 4, 8) of the manuscript, a regression constant and missing values in control variables.

Dependent variables are dummies for: (1) Complies with all out of 9 measures, (2) Avoiding contact with at-risk groups (elderly individuals, chronically ill individuals, (3) avoiding public transport, (4) avoiding trips, (5) avoiding shopping at peak hours, (6) staying away from crowds, (7) keeping a distance from symptomatic people, (8) avoiding touching, such as handshaking or hugging, (9) regularly washing hands, (10) wearing masks.

Table A.5
Robustness checks with survey weights and augmented sample

Outcome:	9 component index (incl. mask)	8 component index (excl. mask)	8 component index (excl. mask)	9 component index (incl. mask)
Sample:	2020: Tranches 2-4	2020: Tranches 1-4	2020: Tranches 1-4	2021
Weighted:	w/ weights	w/o weights	w/ weights	w/ weights
	(1)	(2)	(3)	(4)
Satisfaction with Covid-19 crisis management of German federal government (0 low - 10 high)	3.555***	3.100***	2.913***	1.256*
	(1.100)	(0.687)	(0.893)	(0.721)
Same control variables as in main results (Table 3, cols. 4, 8)	Yes	Yes	Yes	Yes
Observations	3,379	4,973	4,973	5,795
Underidentification: Kleibergen-Paap rk LM statistic	29.913	63.566	42.495	63.543
p-value Kleibergen-Paap rk LM	0.000	0.000	0.000	0.000
Weak identification: Kleibergen-Paap rk Wald F statistic	8.831	20.372	12.979	22.296
Overidentification: Hansen J statistic	7.703	12.515	4.122	6.030
p-value of Hansen J	0.261	0.051	0.660	0.420
Dependent variable mean	0.873	0.911	0.911	0.879

Source: SOEP-CoV waves 1, 2 and SOEP-Core of Socio-Economic Panel (SOEP) (v37).
Standard errors clustered at district-level in parentheses. Coefficients and SEs multiplied with 100 for presentation. * $p < 0.10$, ** $p < 0.05$, *** $p < 0.01$.
Excluded instruments: 7 dummy variables for political party preference. All models control for the same set of control variables as in our main results (Table 3, cols. 4, 8) of the manuscript, a regression constant and missing values in control variables.
